# Supplementary material for: Waveguide holography for 3D augmented reality glasses
Source: Nat Commun. 2024 Jan 2;15:66. doi: 10.1038/s41467-023-44032-1 (PMC10762208; doi:10.1038/s41467-023-44032-1)
Supplement: Supplementary file 1 — Supplementary Information [file 41467_2023_44032_MOESM1_ESM.pdf]

Waveguide holography for 3D augmented reality glasses – Supplementary Material

Changwon Jang[1,+,*], Kiseung Bang[1,+], Minseok Chae[2], Byoungho Lee[2], and Douglas Lanman[1]

[1]Reality Labs Research, Meta, Redmond, 98052, WA, United States
[2]Seoul National University, Seoul, 08826, Republic of Korea
[*]changwon.jang@meta.com
[+]these authors contributed equally to this work

S1 Numerical simulation model of wave propagation in pupil replicating waveguide

Diffractive EPE waveguide can be designed with different types of gratings such as SRG[1–4], volume Bragg gratings[5,6], and polarization dependent gratings[7–10]. Each type may be implemented as reflection type or transmission type, or often both modes can be utilized at the same time. Physically rigorous simulation method of light interaction in waveguide varies depending on the types and configurations of the gratings and typically they are computationally prohibited for large scale simulations. Often, approximation to geometrical optics regime can be useful for the system level design of waveguide displays[11,12], however, coherent interaction must be considered in our study. Thus, we formulate simplified numerical simulation model in wave optics regime by making some assumptions. First, thickness of grating is assumed to be negligible as typical grating has micrometer scale thickness. Second, only the first order interaction is considered meaning that multi-order diffraction or non-TIR Fresnel reflection of light is neglected. Lastly, polarization of light is not considered since SLM is active to only a single polarization and we put a linear polarizer at the out-coupler of the waveguide, matching the SLM polarization direction. Some light is lost at the out-coupler due to the polarization state changes inside the waveguide, however it improves the contrast of the hologram. For simplicity, we assume the waveguide consists of three SRG gratings; input coupler grating, EPE grating, and output coupler grating. Although, the simulation method can be applied to different designs with different shapes, numbers, and arrangement of gratings with a simple modification. Let the input wavefront $u_{in}(x,y)$ be initially in-coupled into the waveguide and guided as total internal reflection mode u_g:

$$u_{g|m=0} = u_{in}\eta_{in}A_{in}e^{k_{gx1}x+k_{gy1}y}, \tag{1}$$

where m is the number of pair of TIR reflection that wavefront has undergone, A_{in} is the aperture function of the input coupler grating, k_{gx1} and k_{gy1} are the momentum of the input grating, and η_{in} represents the coupling efficiency simplified as a single scalar value. Note that the diffraction efficiency can be represented as a function of angular component of the wavefront to simulate more accurate diffraction efficiency. The coupled wavefront bounces back to the grating surface every two total internal reflection mode propagation:

$$u_{g|m=1} = u_{g|m=0} \otimes h_{ASM}, \tag{2}$$

where h_{ASM} is a wave propagation kernel of distance $2t$ in the substrate and $\otimes$ denotes 2D convolution. Note that TIR does not invert the phase of the wavefront. Without losing generality, the m-th TIR propagation from input coupler can be described as follows (m>1):

$$u_{g|m+1} = u_{g|m} \otimes h_{ASM} - u_{g2|m}e^{-j(k_{gx2}x+k_{gy2}y)} - u_{recoupled|m}e^{j(k_{gx1}x+k_{gy1}y)}, \tag{3}$$

where

$$u_{g2|m} = u_{g|m} \otimes h_{ASM}\eta_{epe}M_{epe}e^{j(k_{gx2}x+k_{gy2}y)}, \tag{4}$$

$$u_{recoupled|m} = u_{g|m} \otimes h_{ASM}\eta'_{in}M_{in}e^{-j(k_{gx1}x+k_{gy1}y)}. \tag{5}$$

$u_{g2|m}$ is the diffracted light from EPE grating that is guided to different direction and propagate towards the output coupler grating. Depending on the layout of waveguide, some portion of light $u_{recoupled|m}$ may be re-coupled as -1 order diffraction of the input grating and exit the waveguide with diffraction efficiency η'_{in}. In practical, the coupling efficiency for -1 order can be designed to be smaller than 1st order. For simplicity, we neglect $u_{recoupled}$ and assume EPE mask to be homogeneous, then we can re-write u_g as:

$$\begin{aligned} u_{g|m+1} &= u_{g|m} \otimes h_{ASM}(1-\eta_{epe}) \\ &= u_{g|m} \otimes h_{epe}. \end{aligned} \tag{6}$$

Then the input wavefront of the EPE grating can be rewritten as:

$$\begin{aligned} u_{in2} &= u_{g|1} + u_{g|2} + u_{g|3} + u_{g|4} + \cdots \\ &= u_{in}\eta_{in}e^{j(k_{gx1}x+k_{gy1}y)} \otimes (h_{epe} + h_{epe}^{\otimes 2} + h_{epe}^{\otimes 3} + h_{epe}^{\otimes 4} + \cdots) \\ &= u_{in}\eta_{in}e^{j(k_{gx1}x+k_{gy1}y)} \otimes h'_{epe}, \end{aligned} \tag{7}$$

where $h_{epe} = h_{ASM}(1-\eta_{epe})$ and $^{\otimes n}$ indicates $n-1$ successive convolution of itself. Similarly, we can write input wavefront of the output coupler grating as:

$$\begin{aligned} u_{in3} &= u_{in2}\eta_{epe}e^{j(k_{gx2}x+k_{gy2}y)} \otimes (h_{out} + h_{out}^{\otimes 2} + h_{out}^{\otimes 3} + h_{out}^{\otimes 4} + \cdots) \\ &= u_{in2}\eta_{epe}e^{j(k_{gx2}x+k_{gy2}y)} \otimes h'_{out}, \end{aligned} \tag{8}$$

where $h_{out} = h_{ASM}(1-\eta_{out})$. And finally, the output wavefront is represented as:

$$u_{out} = u_{in3}\eta_{out}e^{j(k_{gx3}x+k_{gy3}y)} \tag{9}$$

where k_{gx3} and k_{gy3} are the momentum of the output coupler grating. In the typical design of EPE waveguide $k_{gx1} + k_{gx2} + k_{gx3} = 0$ and $k_{gy1} + k_{gy2} + k_{gy3} = 0$, which leads to:

$$u_{out} = u_{in} \otimes h_{wg} \tag{10}$$

where

$$h_{wg} = \mathscr{F}^{-1}(\eta_{in}\eta_{epe}\eta_{out}H'_{epe}(f_x - k_{gx1}/2\pi, f_y - k_{gy1}/2\pi) \times H'_{out}(f_x - k_{gx2}/2\pi, f_y - k_{gy2}/2\pi)). \tag{11}$$

Equation 11 implies that the coherent interaction in the waveguide can be simply represented as a complex valued kernel h_{wg} by neglecting multi-order diffraction and the clipping effect at the grating boundaries. This result offers a physical intuition that the waveguide can be closely approximated as an LSI system. Also, the result can be utilized for useful numerical simulations presented in the design space analysis section since the computation can be significantly reduced compared with full tracking of light path that requires numerous convolution process. Although, more precise simulation can be performed based on Eq. 1-5 without applying assumptions. To validate the single kernel approximation, we have trained the model with simulated wavefront dataset without approximation and compared the fidelity. In the simulation, we added some non-idealities of the system such as DC noise and slight aberration. With the single kernel, 24.7 dB of c-PSNR was achieved, while 9-channel model could achieve 27.1 dB. We use the result of Eq.11 for design space and scalability analysis presented in our work.

S2 Analytic derivation of gradient in the waveguide model

We provide the analytic derivation of the waveguide model that can be used for model training. The estimated output of the waveguide model u' can be represented as:

$$u' = \mathscr{A}(u_{in}) = \left[\sum_i \{(u_{in} \times Q_i) \otimes h_i\} \times R_i \right] + DC, \tag{12}$$

where $\mathscr{A}$ is the waveguide model , u_{in} is the input field. u_{in} and Q_i are $N \times N$ complex valued matrices, u',R_i, and DC are $M \times M$ complex-valued matrices, and h_i is $(N+M) \times (N+M)$ complex valued matrix. $\otimes$ denotes a non-circular convolution operation defined in the input and output domain of $N^2, (N+M)^2 \rightarrow M^2$. Generally, discrete convolution operations can be calculated using fast Fourier transform (FFT), which has lower order of time complexity. However, input and output domain

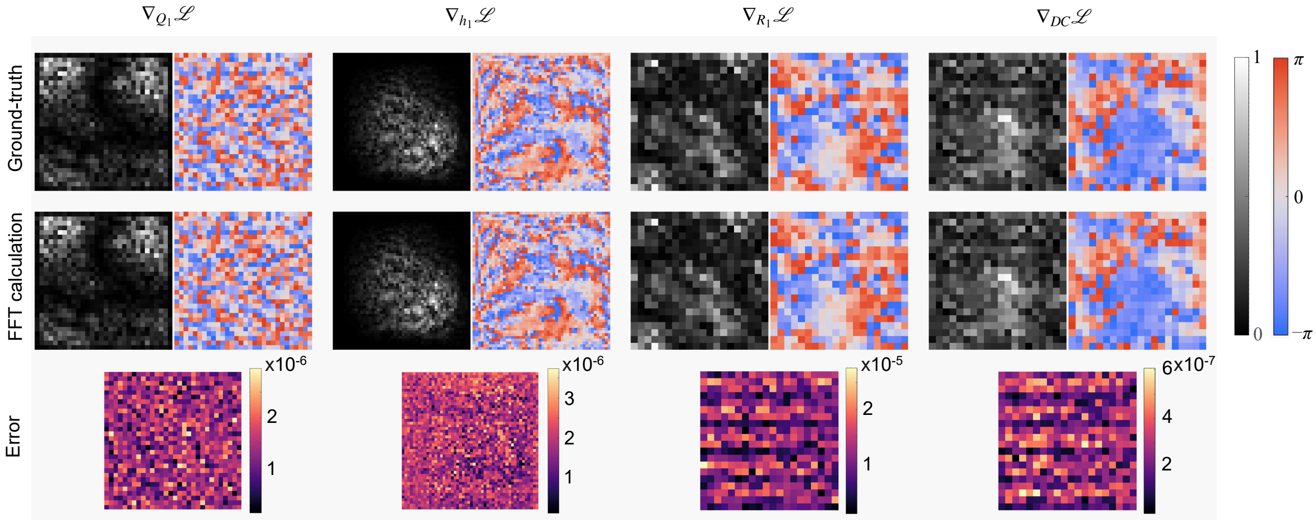

Supplementary Figure 1. Example of gradients for each modeling parameter for validation of Eq. 15-18. From the left, $\nabla_{Q_1}\mathscr{L}$, $\nabla_{h_1}\mathscr{L}$, $\nabla_{R_1}\mathscr{L}$, and $\nabla_{DC}\mathscr{L}$ are represented. The amplitude of the gradients was normalized to unity. The top row shows the ground-truth gradients, the middle row shows the gradient calculated using FFT as shown in Eq. 15-18, and the bottom row shows the norm of the complex error between the ground-truth and the FFT calculation. The ground-truth was obtained with a small-step change for each pixel. The FFT calculation result matches the ground-truth correctly with only a negligible difference, which is caused by the finite step change in ground-truth calculation. All the illustrated parameters are down-sampled from the original size for visualization.

size in FFT should be same and it gives circular convolution result. Therefore, to calculate the convolution $\otimes$ with FFT, it should be applied in modified way using additional zero-padding and crop operators.

$$u' = \mathscr{A}(u_{in}) = \left[\sum_i \mathscr{P}_{(N+M)^2\to M^2}\left[\mathscr{F}^{-1}\left\{\mathscr{F}\left(\mathscr{P}_{N^2\to(N+M)^2}(u_{in}\times Q_i)\right)\times\mathscr{F}(h_i)\right\}\right]\times R_i\right] + DC \tag{13}$$

$\mathscr{F}$ denotes the 2D FFT defined in the input and output domain of $(N+M)^2, (N+M)^2 \to (N+M)^2$. $\mathscr{P}_{A\to B}$ is the zero-padding and crop operator where A and B is the size of input and output. When $A < B$, it operates as a zero-padding operator, and when $A > B$, it operates as a crop operator around the center.

The cost function for model accuracy is defined as follows:

$$\mathscr{L} = \|u' - u\|^2, \tag{14}$$

where u is the captured output $M\times M$ complex wavefront matrix. While optimizing the model, we update Q_i, h_i, R_i, and DC, so we need the gradient of the cost function for each parameter. Each gradient can be calculated using the FFT as follows:

$$\nabla_{h_i}\mathscr{L} = -2\mathscr{F}^{-1}\left[\overline{\mathscr{F}\left\{\mathscr{P}_{N^2\to(N+M)^2}(u_{in}\times Q_i)\right\}}\times\mathscr{F}\left\{\mathscr{P}_{M^2\to(N+M)^2}\left(\overline{R_i}\times(u'-u)\right)\right\}\right], \tag{15}$$

$$\nabla_{Q_i}\mathscr{L} = -2\mathscr{P}_{(N+M)^2\to N^2}\mathscr{F}^{-1}\left[\overline{\mathscr{F}\{h_i\}}\times\mathscr{F}\left\{\mathscr{P}_{M^2\to(N+M)^2}\left(\overline{R_i}\times\left(u'-u\right)\right)\right\}\right]\times\overline{u_{in}}, \tag{16}$$

$$\nabla_{R_i}\mathscr{L} = -2\overline{\mathscr{P}_{(N+M)^2\to M^2}\mathscr{F}^{-1}\left[\mathscr{F}\{h_i\}\times\mathscr{F}\left\{\mathscr{P}_{N^2\times(N+M)^2}(u_{in}\times Q_i)\right\}\right]}\times\left(u'-u\right), \tag{17}$$

$$\nabla_{DC}\mathscr{L} = -2(u'-u), \tag{18}$$

where the gradient of a real-valued function g in the complex domain is defined as $\nabla_{a+ib}g = \nabla_a g + i\nabla_b g$, when a and b are real numbers, and $\overline{(\cdot)}$ is the complex conjugation operator. The correctness of Eq. 15-18 is demonstrated in Supplementary Fig. 1 through the comparison with the ground-truth result. Although we utilized the auto-gradient functionality of Pytorch[13] for the flexibility of the modeling, direct access to gradient calculations of Eq. 15-18 could enable further acceleration of computation in the future.

S3 Wavefront camera calibration algorithm

The wavefront camera is built based on Mach-Zehnder type interferometer that consists of a beam splitter, a piezo actuator (PZ-38), a neutral density filter, and a CMOS sensor. The sensor captures the interference pattern generated by the signal wavefront and the plane reference wavefront, with shifted phase controlled by the piezo actuator. In order to compensate the phase fluctuation caused by airflow and vibration, the piezo actuator is updated using phase locking algorithm and minimize the phase error to get correct interference pattern with target phase shift. The pre-calibration of the interferometer system is useful for achieving the precise phase locking during the wavefront data acquisition. The details of the pre-calibration and phase locking algorithm is described in the following paragraph.

a **b**

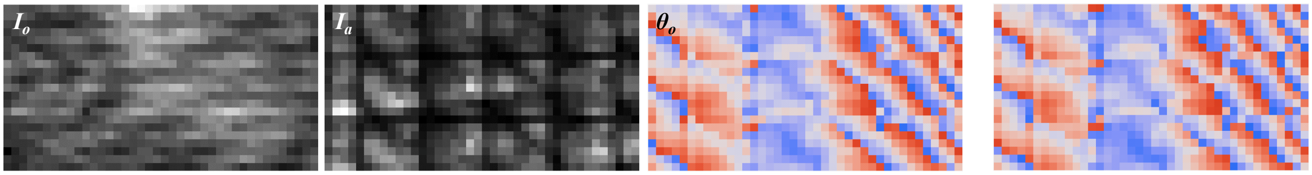

Supplementary Figure 2. Example of pre-calibration result of Eq.19. **a** Illustration of I_o, I_a and θ_o after the pooling process. **b** A sample phase of output wavefront after the pooling process captured during the dataset acquisition, which shows a very similar phase profile as θ_o in **a**.

With the fixed signal wavefront and phase-shifted reference wavefront, the intensity of the interference pattern follows a sinusoidal function of the phase shift ϕ_s.

$$I(x,y;\phi_s) = I_o(x,y) + I_a(x,y)\cos\left(\theta_o(x,y)+\phi_s\right) \tag{19}$$

In the pre-calibration stage, we capture multiple interferograms with unknown phase shift ϕ_s varying the piezo-actuator and find the optimal values of the pre-calibration parameters such as intensity offset I_o, intensity amplitude I_a, and phase offset θ_o that closely approximate Eq. 19. With pre-calibration parameters, the unknown phase shift ϕ_o in the data acquisition stage can be estimated with a fairly high accuracy when arbitrary interferogram I_{cap} is acquired. We can quantify the accuracy of the estimation by the cost function g and use it to find the estimated phase shift ϕ_e.

$$g = \|I_{cap} - I(x,y;\phi_s)\| \tag{20}$$

$$\phi_e = \text{argmin}_{\phi_s} g \tag{21}$$

With the knowledge of the cost function g being a sinusoidal function of ϕ_s, a simple binary search can result in a quick and accurate estimation. Using the estimated phase, we update the piezo actuator to be matched to the target phase shift.

We also used the value of cost function to evaluate the perturbation in the system, which helped to get high-quality dataset for the waveguide modeling.

Note that above phase locking algorithm is assuming that the signal wavefront remains the same in both the calibration and estimation stage. In practice, the SLM pattern needs to be updated as multiple random phases during the data acquisition process. In order to provide the robustness to the algorithm, average-pooling process is applied to the captured interference pattern with a pooling area of 100×100, which eliminates the effect of the high frequency components by the random phase input. This allows the system to calibrate for arbitrary fully random phase inputs after I_o, I_a, and θ_o are acquired. Supplementary Fig. 2 illustrates the pre-calibration results of I_o, I_a, and θ_o. After obtaining $N(N>3)$ phase shifted interferograms, a complex wavefront can be reconstructed as follows:

$$u = \frac{1}{N}\sum_{k=1}^{N} I_k \exp\left(i\frac{2\pi k}{N}\right) \tag{22}$$

We built two wavefront cameras in the benchtop; one for capturing the relayed SLM image and the other for capturing waveguide output wavefront. Captured data of relayed SLM wavefront is used to calibrate the physical propagation module that can be set as an initialization for waveguide model training.

S4 Details of physical propagation module

Ideally, the phase response of all SLM pixels is linear to the gray level (or voltage) and covers the full modulation range of $-\pi$ to $+\pi$. However practically, the phase response is not linear, nor consistent over the SLM area. To calibrate the phase response of the SLM, we inserted a gamma function layer as follows.

$$\phi_{out}(x,y) = \sum_{i=0}^{3}\sum_{j=0}^{3}\sum_{k=0}^{3} c_{ijk} \times x^i \times y^j \times \phi_{in}(x,y)^k, \tag{23}$$

where x and y are the position of the pixel, and ϕ_{in} and ϕ_{out} are the input phase (gray level) and the output phase. c_{ijk} is the set of coefficients that is adjusted for calibration. When $c_{ijk} \neq 0$ for $i, j > 0$, the model allows spatially-varying nonlinear SLM response. A similar calibration method and result have been reported recently[14].

Crosstalk of the spatial light modulator

It is known that the liquid crystal on silicon panel with a small pixel pitch suffers from the field fringe effect.[15] When the voltage is applied to a LC cell in a pixel, the effective electric field is disturbed by the leaked electric field from the adjacent pixels. To include such field fringe effect, we inserted a 3×3 convolution layer after the gamma function layer. The convolution is applied in a real domain of the phase after upsampling layer with a factor of two, before converted to the complex wavefront. The upsampling is performed because the field-fringe effect noise includes a higher frequency components that cannot be captured by the maximum spatial frequency supported by the SLM pixel pitch.

Homography mapping function

To match the coordinates between the SLM pixels and the camera pixels and also compensate for the distortion, we re-map the homography of the wavefront before applying the optical propagation. We used up to 2nd order coefficients of homography transformation using 12 parameters. Similar functionality can be implemented by piece-wise affine transform[16].

$$\begin{bmatrix} x' \\ y' \end{bmatrix} = \begin{bmatrix} a_{20} & a_{11} & a_{02} & a_{10} & a_{01} & a_{00} \\ b_{20} & b_{11} & b_{02} & b_{10} & b_{01} & b_{00} \end{bmatrix} \begin{bmatrix} x^2 \\ xy \\ y^2 \\ x \\ y \\ 1 \end{bmatrix} \tag{24}$$

Optical propagation and tilt

Band-limited angular spectrum method[17] is used for numerical propagation in the model. The angle alignment error between the SLM and waveguide can be also considered in the model by using 3D tilt propagation[18], however the computation involves re-sampling process which adds a slight error. Therefore we did not include it in the final result of the benchtop prototype calibration. The propagation parameter is pre-calibrated using a wavefront camera that images a relayed SLM and fixed during the waveguide model training stage.

S5 Details of Optical Architecture

S5.1 Design parameters of pupil-replicating waveguides

The design of our waveguide shares general goals with conventional pupil-replicating waveguides, including high light throughput efficiency, a compact form factor, and uniform out-coupled light intensity across both eyebox domain and field of view domain. Due to numerous design parameters intertwined together, the design space becomes multi-dimensional, with many trade-off relations to consider. For convenience, we can divide the design parameters into three important groups as follows: layout of the waveguide (shape, size, and position of each grating), substrate selection (refractive index and thickness), and grating parameters (grating pitch and structure of surface relief grating). The following is a high-level description of our waveguide design process. For the layout, we adopted the design introduced by Tapani Levola[1,2], which consists of an in-coupler, an exit-pupil expanding grating (EPE grating), and an out-coupler. Since introduced, this design is now widely used in industry and academia. The out-coupler size is designed to cover the target eyebox size of 16 × 12 mm, which is considered large enough for near-eye displays. Then the trapezoid shape of EPE coupler is designed to deliver the target field of view efficiently, based on the simulated ray trajectory. We set the input coupler size comparable to user's pupil size, to match the information amount of the input and target output pupil. For the waveguide substrate, refractive index and thickness should be selected. Thickness is an important parameter that affects the density of pupil replication and the form factor.

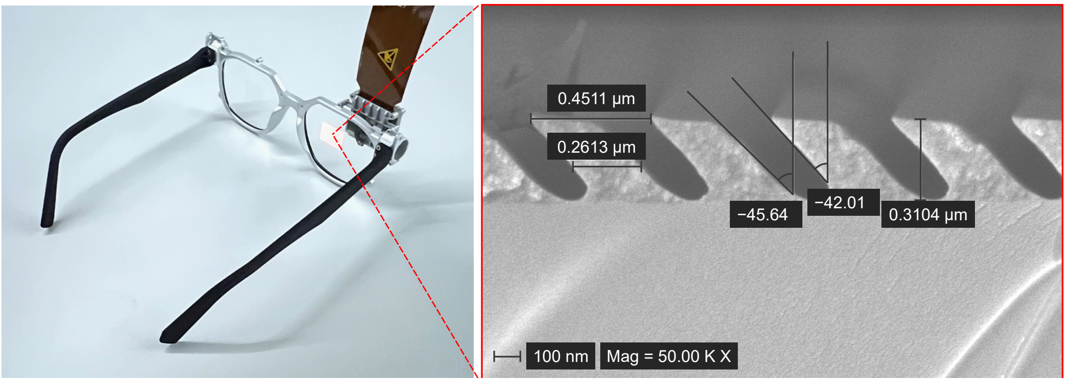

Supplementary Figure 3. A scanning electron microscope photograph of waveguide sample used in the prototypes.

Generally, having a high refractive index is desired because it results the larger TIR bandwidth (the range of possible modes that can be transmitted via total internal reflection), as well as it reduces unwanted artifacts such as visible diffracted light from world side. However, the refractive index cannot be arbitrarily selected because of practical limitations such as material availability, cost, and fabrication methods. Hence, we used a standard glass material (n=1.5) for our project. Designing gratings involves determining the pitch, aspect ratio, and shape of the grating structure. The pitch determines the distance of pupil replication and affects the density of replication, along with the substrate thickness. The structure of grating is designed using coupled wave analysis (RCWA) or finite-difference time-domain method to optimize the efficiency and selectivity of diffraction. Supplementary Fig. 3 shows the grating structure of the waveguide used in our work.

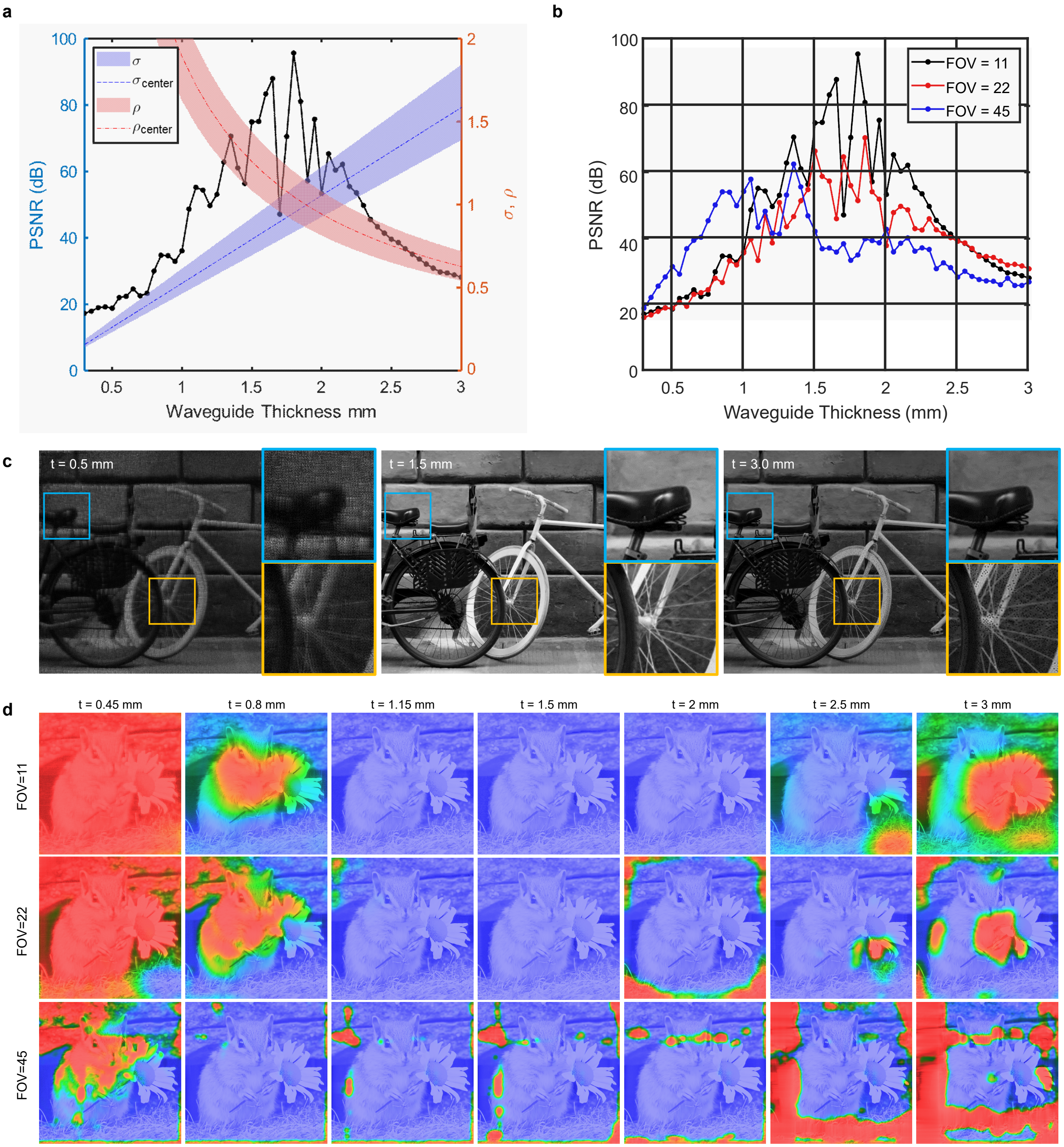

Supplementary Figure 4. **a** Simulated display performance sweeping the waveguide thickness while other system parameters remain identical as the benchtop prototype. The display performance is evaluated as an average PSNR value of five different images simulated to be displayed at 1 m. the pupil density ρ and its inverse σ are plotted together. **b** Simulated display performance plot varying the field of view to evaluate the scalability of the system. The pixel pitch is set as 4 µm, 2 µm, and 1 µm respectively, from the smallest FOV. For 1 µm pixel pitch, the refractive index of waveguide is set as 2 to support the full field of view. **c** Simulated display results with different waveguide thickness, The artifacts are demonstrated when the waveguide thickness is too thin or too thick. Bicycle image by Fiore Power (CC BY 2.0).**d** Visualization of field of view uniformity according to varying waveguide thickness and FOV, using pseudo colormap of hdrvdp2 metric[19]. The red area indicates that the observer would perceive the artifact with a high probability compared to the original image.

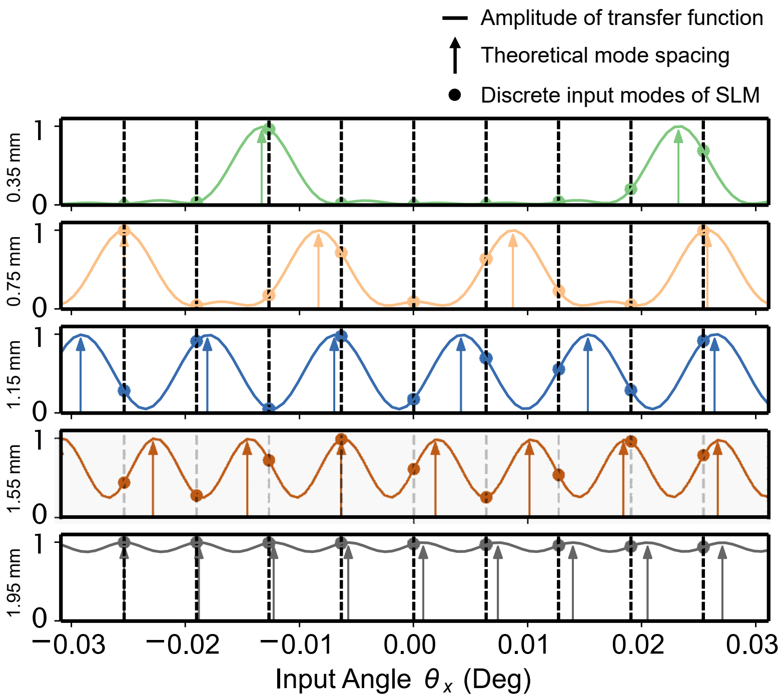

Supplementary Figure 5. **a** Illustration of the mode spacing characteristics of pupil-replicating waveguides. Amplitude of the transfer function is calculated as a ratio of input and output energy transmitted through the pupil-replicating waveguide corresponding to its angular component, simulated based on S1. Note that the peak of simulated transfer function matches the theoretical mode spacing of ideal waveguide, while both are calculated separately. The product of discrete angular components of input field from the SLM and the transfer function predicts the signal loss that affects the degree of freedom of wavefront optimization.

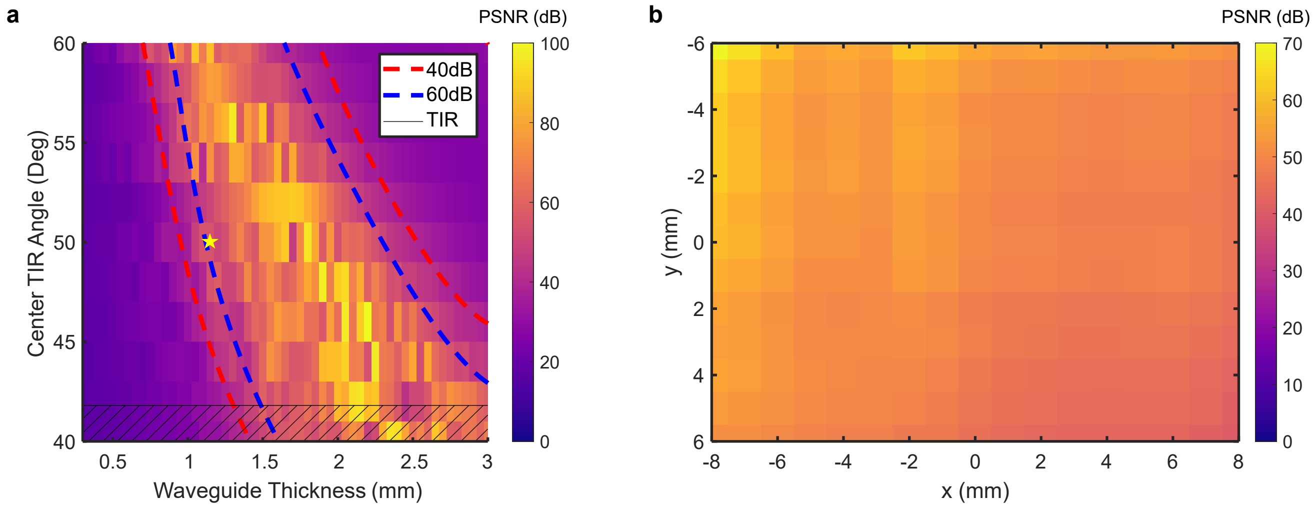

Supplementary Figure 6. **a** Visualization of the design space. Our design choice is indicated as a yellow star in the plot. With the selected substrate material and TIR angle, we use 60 dB contour line as a criteria while minimizing the thickness as thin as possible. **b** Visualization of the eyebox uniformity simulated with our design choice.

S5.2 Design space and scalability analysis

Among the various parameters involved in the design space, we focus on pupil replication distance d_{rep} which is a function of thickness t and the TIR angle θ_{TIR} of corresponding field of view component as:

$$d_{rep} = 2t \tan \theta_{TIR}. \tag{25}$$

If d_{rep} is set too large, some field of view components may not fill the eye pupil and cause vignetting artifact or partial loss of the image. Otherwise, when the pupil replication distance is too dense, the intensity of guided light decays too fast because it requires too many TIRs for the light propagation over the entire eyebox. In the conventional waveguide displays, the resolution tends to be degraded with thinner waveguide as effective numerical aperture is reduced and more clipping happens. In waveguide holography, the numerical aperture is not necessarily degraded as demonstrated in the previous section. However, it imposes a different restriction in the wavefront optimization. If the wavefront is replicated and overlapped too densely, it reduces the degree of freedom to control the interference in a desired manner, which trades the image quality. We can define the replication density ρ and degree of freedom for each k-vector (or FOV) component σ as follows:

$$\rho = S_{in}/d_{rep} \tag{26}$$

$$\sigma = d_{rep}/S_{in}. \tag{27}$$

where S_{in} is the smaller of the exit pupil size of the projector or entrance pupil size of the waveguide. The absolute value of each does not necessarily predict the performance when it is close to or larger than 1, but if the value become too small, it is expected to negatively impact the image quality. The simulation results presented in Supplementary Fig. 4**a** show that there is a range of sweet spot that achieves the good image quality, where d_{rep} is balanced between pupil density and the wavefront optimization freedom.

Physically, the degree of freedom for wavefront optimization can be interpreted in the guided-wave optics regime when d_{rep} is small. As described in Eq. 2 in the manuscript, the theoretical mode spacing of the waveguide is determined as $\delta\theta_{res} = \lambda/2t \tan \theta_T$. In the ideal case of boundary/loss-less waveguide with an infinite input beam size, only discrete angular modes can be transmitted through the waveguide. However, in practical pupil-replicating waveguides with leaky gratings and physical boundaries, the transfer function of the waveguide becomes quasi-continuous with varying amplitude. When this effect is combined with discrete angular mode spacing of input wavefront generated from pixelated SLM, it can be predicted that some angular components of the input field will be lost during the waveguide propagation, as shown in Supplementary Fig. 5. As the thickness becomes thinner and d_{rep} decreases, this signal loss will negatively affect the optimization freedom for the output wavefront. It could also explain the ringing in Supplementary Fig. 4**a** as a result of Moiré effect between the transfer function of the waveguide and the discrete input field from the SLM. However, the typical mode spacing of waveguide is dense enough for human visual acuity, and some loss would be tolerable without noticeable degradation.

Supplementary Fig. 4**b** shows the scalability in terms of field of view varying the pixel pitch of the SLM. As the supported field of view increases, the maximum PSNR tends to decrease slightly; however, a PSNR over 40 dB would not make a practical difference in perceived image quality. Supplementary Fig.4**c** illustrates both artifacts related to the thickness in simulation, using the specifications of the benchtop prototype. The perceptual artifact in the field of view domain is visualized in Supplementary Fig. 4**d**, varying the system FOV. When the waveguide thickness is too thin, the artifact tends to be dependent on the texture of displayed image since the cause is limited optimization freedom. While the thickness is too thick, the artifact tends to show arbitrary pattern independent to the contents because the vignetting from the pupil density becomes the main limiting factor.

The simulation results provide valuable insights for optimizing the design parameters of the system, as well as demonstrating the architecture's scalability. In Supplementary Fig. 6**a**, we plot the design space of the waveguide guide based on its thickness and center TIR angle. The center TIR angle is selected based on the refractive index of the substrate and the target field of view, which is set to 50 degrees in our case. Based on the simulation results, our design strategy is to simultaneously maximize the PSNR and minimize the thickness of the waveguide. With the selected design, the uniformity of the image quality in the eyebox domain is verified through simulation, as presented in Supplementary Fig. 6**b**. This is an important feature of our software-steered eyebox expansion approach, which distinguishes it from non-replicating waveguides that only work with a small eyebox.

S5.3 Additional Notes on the Architecture

Supplementary Fig. 7 is a photograph of benchtop prototype. In both prototypes, a collimated light source is generated externally as shown in Supplementary Fig. 8**a**.

Due to the limited fill-factor of SLM, a small portion of light is reflected without modulated and generates a DC noise. When a Fourier hologram is displayed, the DC noise forms a bright spot at the center field of view. Typically, a half band filter

is placed in the Fourier domain[20,21] to eliminate the noise. To retain a compact form factor, an angular stop filters[22] can be used without 4-f relay optics as shown in Supplementary Fig. 8**b**.

The FOV of current design is limited by the pixel-pitch of the SLM because we use a direct view hologram projection configuration without projection lens. There are ongoing efforts in the academia and industry to develop and manufacture sub-micrometer pixel pitch panels[23,24] with growing expectation of AR/VR, which will be the long term solution for the FOV. Although, we note that there are alternative variations of hologram projection module to increase FOV immediately. For example, a projection lens can be used similarly to a conventional image projection module as shown in Supplementary Fig. 8**c**, so the FOV is limited by the focal length of the lens and the size of the SLM. In this case, the DC noise is spread in the entire FOV domain. However, such configuration could focus DC noise close to the eye-box domain, that will result non-uniform eye-box quality. In this case, an input-coupler aperture or additional spatial filter can be used to filter the high-order and DC noise as shown in Supplementary Fig. 8**c**. Nevertheless, we choose to showcase the feasibility of the ultimate lens-free architecture, betting on the future breakthrough in the micro display technology.

For further miniaturization, the illumination module can be modified. One potential is to directly illuminate the SLM through the waveguide. However, if the input beam is illuminated through the SRG waveguide, the reverse path at the in-coupler will generate unwanted diffraction term that is mixed with the signal as shown in the left of Supplementary Fig. 8**d**, indicated as $-R1^{st}$. As a solution, polarization volume grating in-couplers can be used to eliminate unwanted diffraction orders due to as shown in the right of Supplementary Fig. 8**d**. Also, as shown in Supplementary Fig. 8**e**, collimated illumination can be generated in a more compact manner using a single mode waveguide or a beam expanding wedge prism[25].

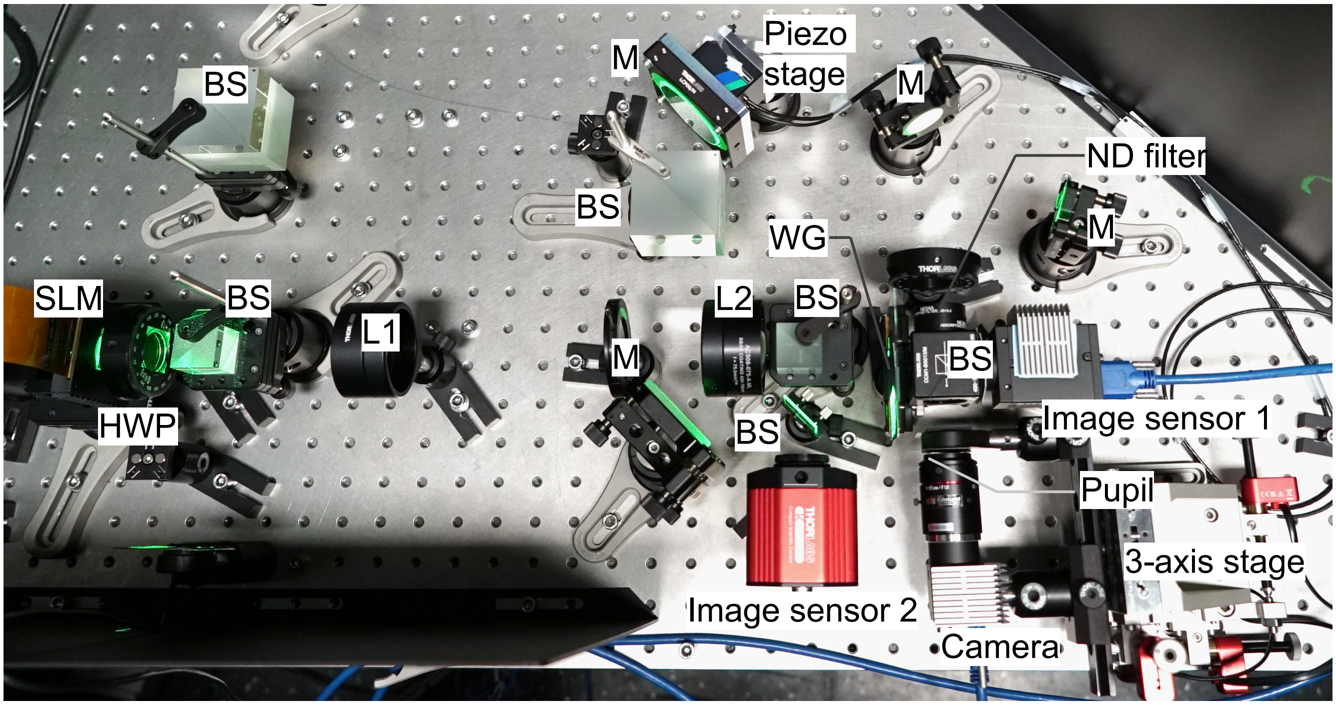

Supplementary Figure 7. A photograph of the benchtop experimental setup.

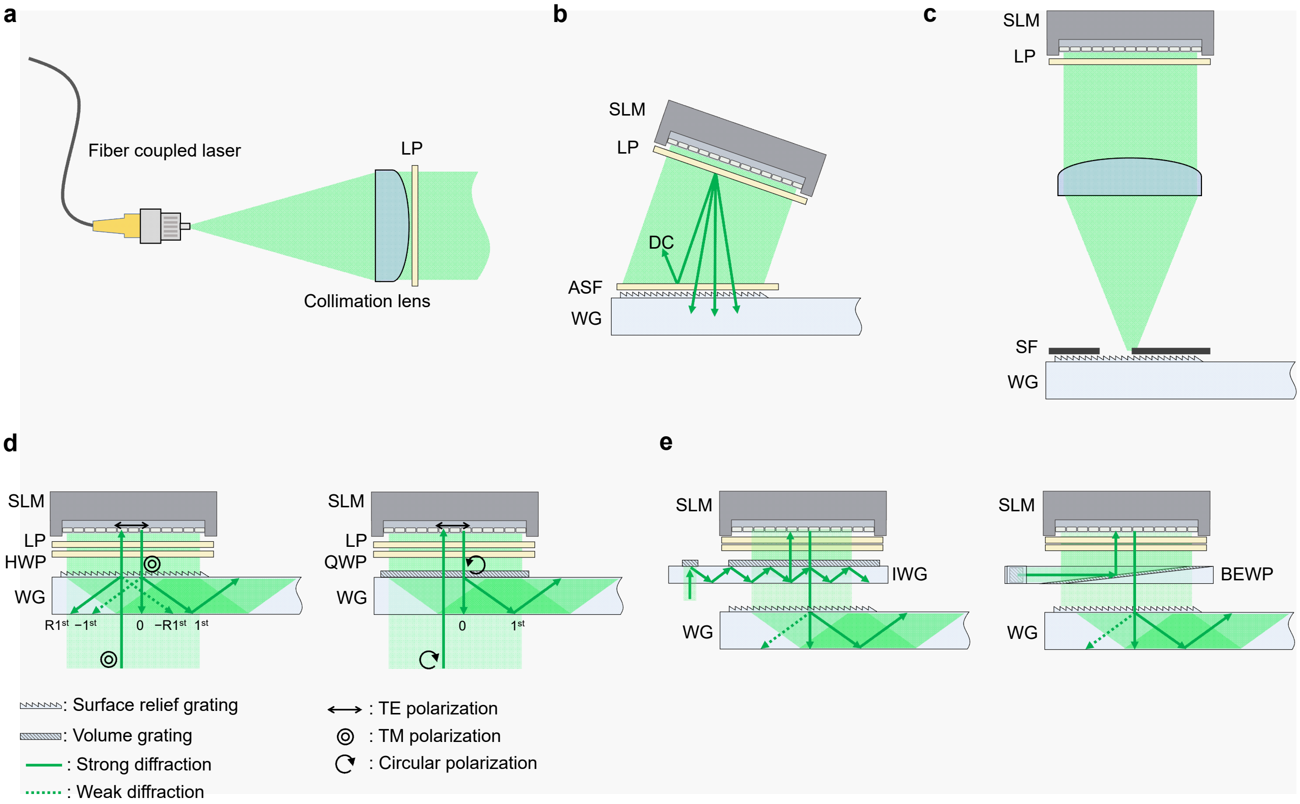

Supplementary Figure 8. Illustrations of further details of the system and potential miniaturization strategies. **a** Photographs of waveguide sample used in the prototypes. **b** Beam expanding system for plane wave illumination. **c** The hologram projection module with a projection lens. **d** Illustrations of hologram projection module without the beam splitter, with respect to type of in-coupler grating. Left figure shows surface relief grating while right figure shows polarization volume grating which does not generate unwanted diffraction. **e** Example designs of miniaturized illumination module. Left figure illustrates illumination waveguide (IWG) and the right figure illustrates beam expanding wedge prism (BEWP)[25].

S6 Additional Results

Supplementary Fig. 9 illustrates the entire model parameters of multi-channel kernels h and complex apertures R that are partially presented in Fig. 3**a** of the manuscript. Supplementary Fig.10 demonstrates the experimental results of the waveguide holography when images are displayed at infinity conjugate. Additional results captured in different pupil locations are provided in Supplementary Figs. 11–13, as proof of eyebox expansion. Relative pupil (x, y, z) locations of pupil 1–6 are $(-4,3,0)$, $(1,1,0)$, $(5,4,0)$, $(-3,-2,2)$, $(-5.5,-4.5,-5)$, and $(3,-2,5)$ respectively, in millimeters. The center of the eyebox is set as 25 mm distance (to normal direction) from the center of the out-coupler grating. Supplementary Fig. 14 illustrates the effect of pupil offset, simulating the eyetracking error during the software-pupil steering. The upper row (cat image) is displayed at 1 diopter and the bottom row (tower image) is displayed at 0 diopter.

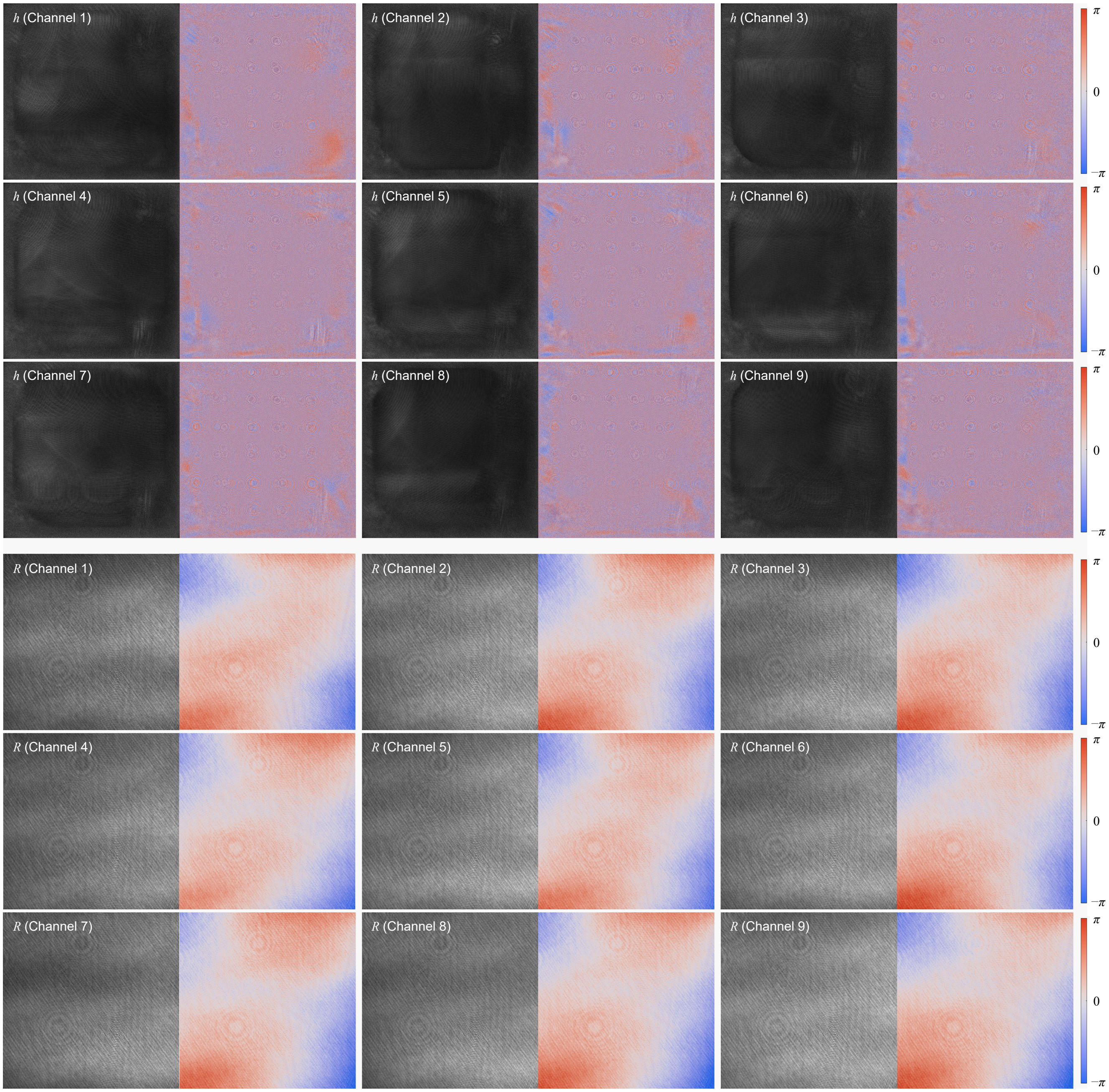

Supplementary Figure 9. Visualizations of optimized model parameters as gray-scale amplitude and pseudo-color map phase images: multi-channel kernels h (top) and multi-channel complex apertures R (bottom).

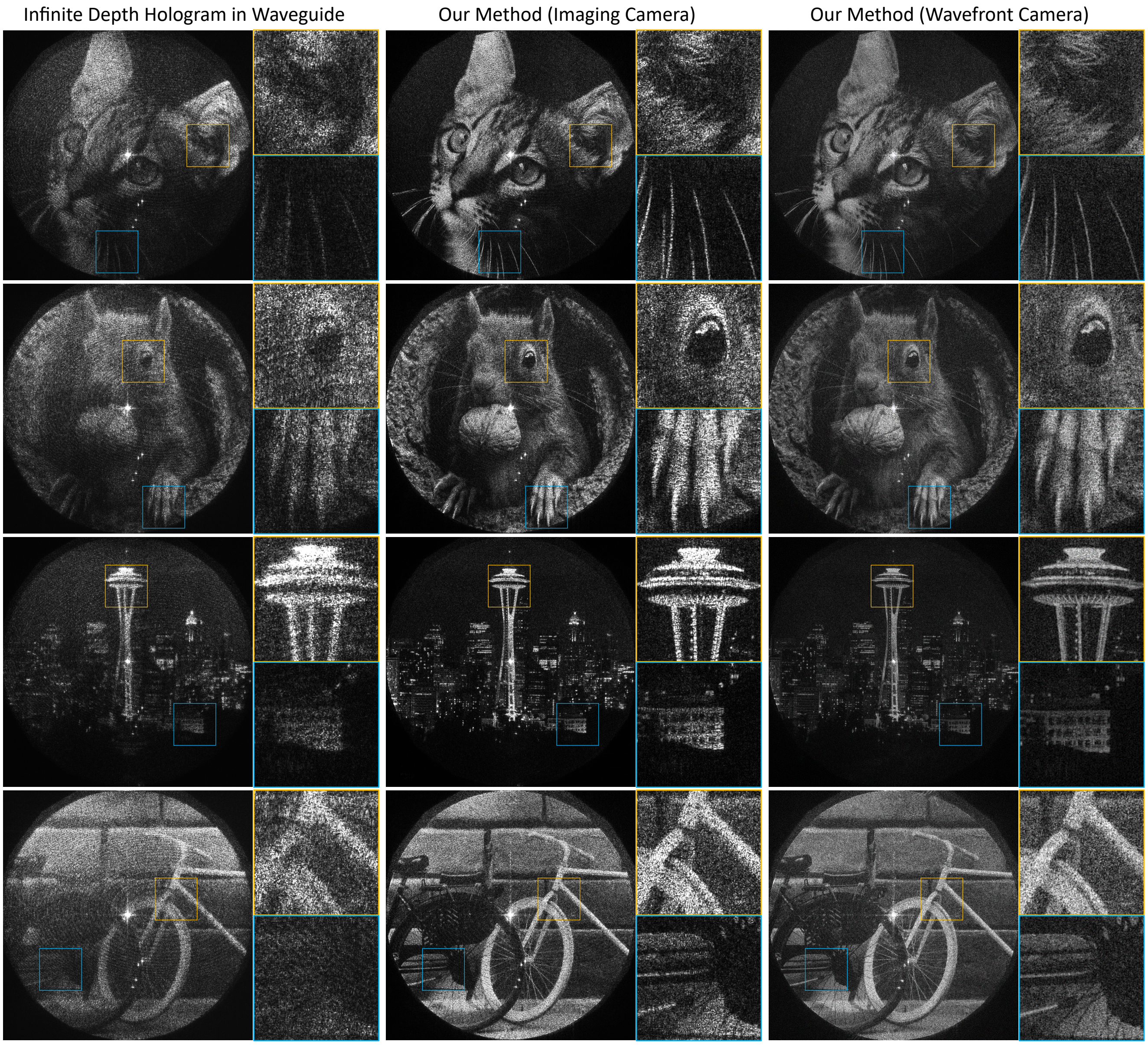

Supplementary Figure 10. Experimental results of waveguide holography when the images are displayed at infinity (0 D). The insets correspond to 1.3 degree of field of view. Our model improves the image quality significantly even when the image is displayed at the infinity depth, where there is no explicit presence of ghost noise. It demonstrates that our method can overcome the non-idealities in the waveguide display system such as surface aberration and beam clipping effect. Cat image by Lali Masriera (CC BY 2.0), Seattle skyline image by fiction-parade (CC BY-SA 2.0), bicycle image by Fiore Power (CC BY 2.0).

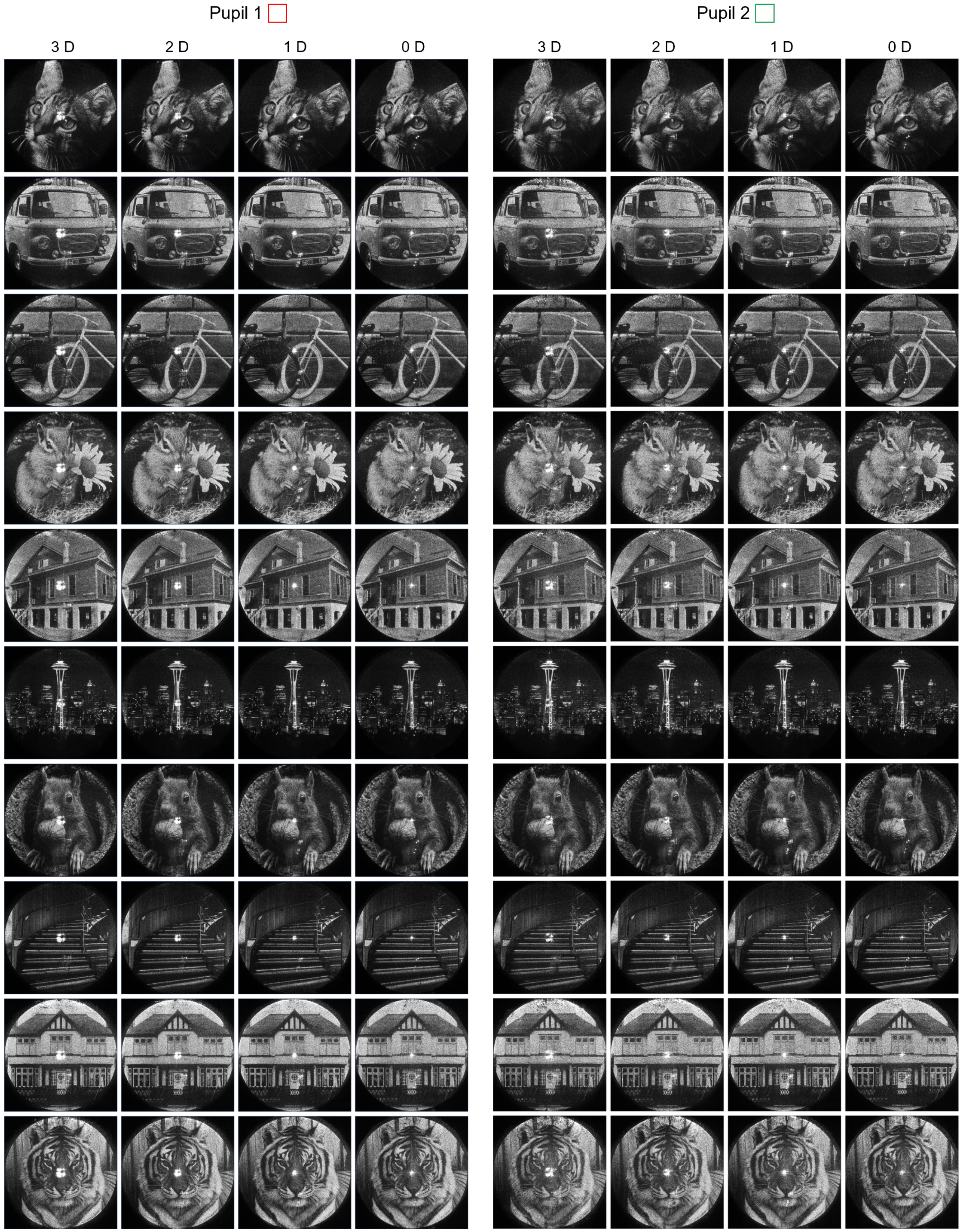

Supplementary Figure 11. Display results at different pupil locations (pupil 1 and pupil 2), captured with imaging camera. Cat image by Lali Masriera (CC BY 2.0), bus image by Farhad Sadykov (CC BY 2.0), bicycle image by Fiore Power (CC BY 2.0), house image by Patrick Feller (CC BY 2.0), Seattle skyline image by fiction-parade (CC BY-SA 2.0), tudor style house image by Nikk (CC BY 2.0).

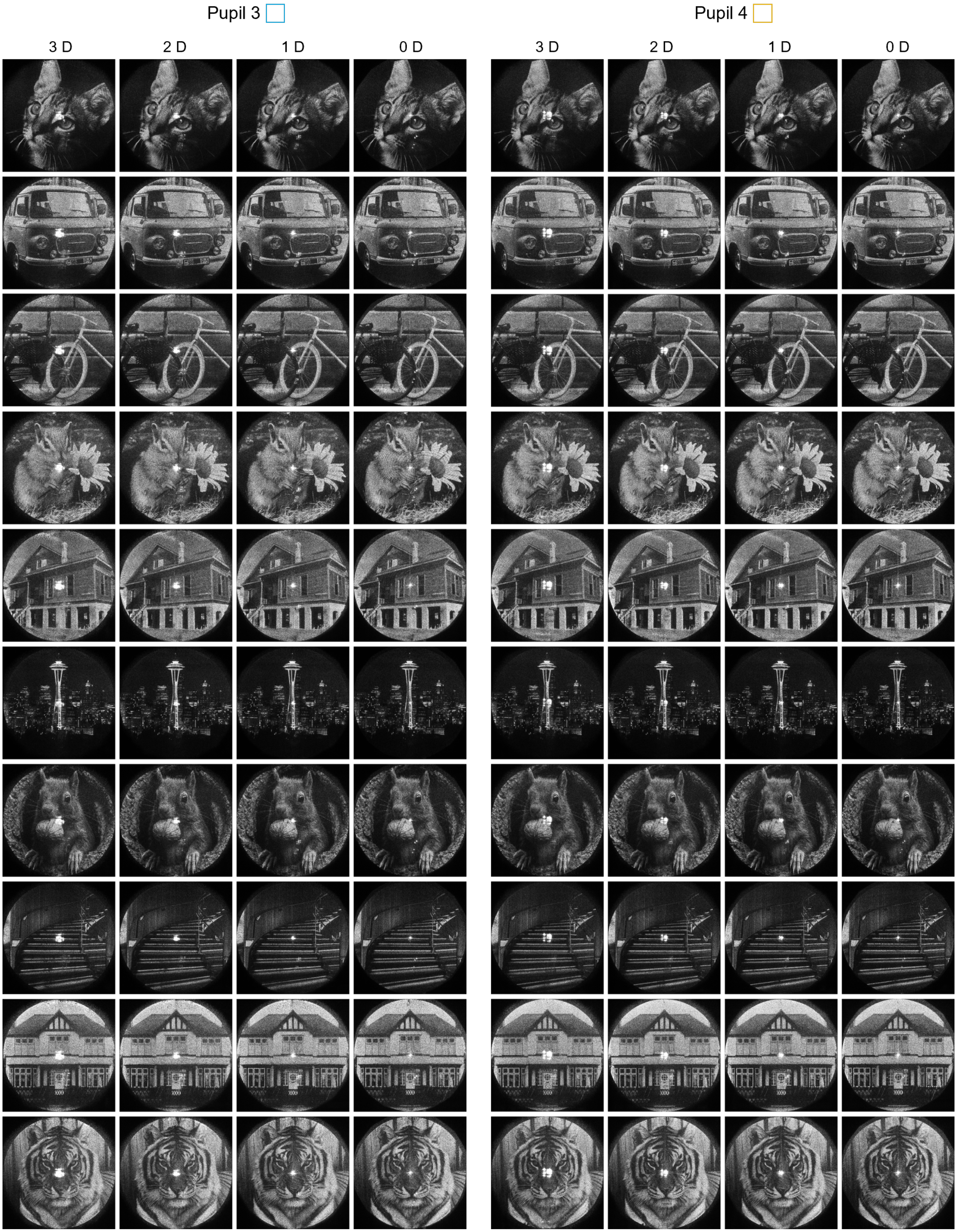

Supplementary Figure 12. Display results at different pupil locations (pupil 3 and pupil 4), captured with imaging camera. Cat image by Lali Masriera (CC BY 2.0), bus image by Farhad Sadykov (CC BY 2.0), bicycle image by Fiore Power (CC BY 2.0), house image by Patrick Feller (CC BY 2.0), Seattle skyline image by fiction-parade (CC BY-SA 2.0), tudor style house image by Nikk (CC BY 2.0).

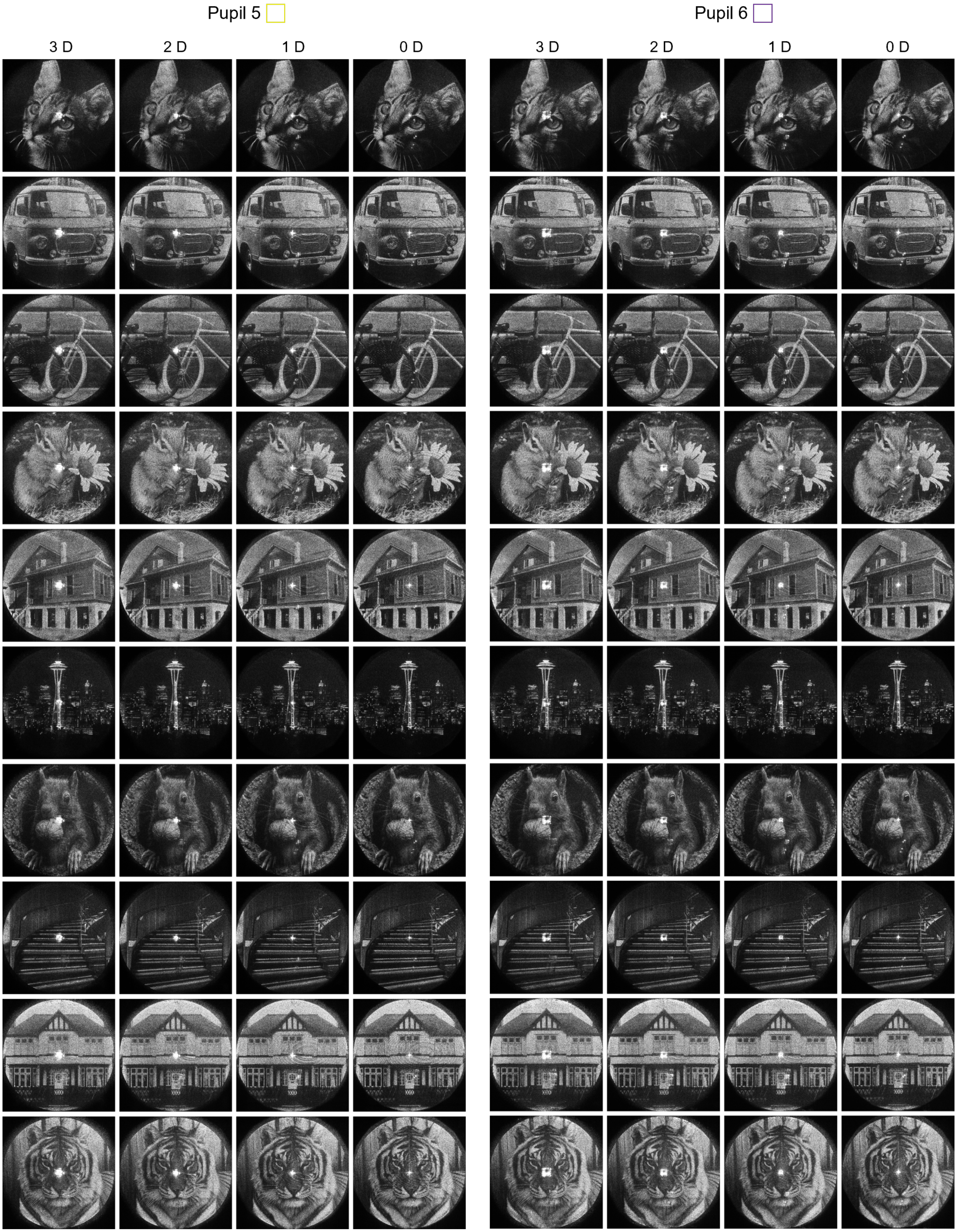

Supplementary Figure 13. Display results at different pupil locations (pupil 5 and pupil 6), captured with imaging camera. Cat image by Lali Masriera (CC BY 2.0), bus image by Farhad Sadykov (CC BY 2.0), bicycle image by Fiore Power (CC BY 2.0), house image by Patrick Feller (CC BY 2.0), Seattle skyline image by fiction-parade (CC BY-SA 2.0), tudor style house image by Nikk (CC BY 2.0).

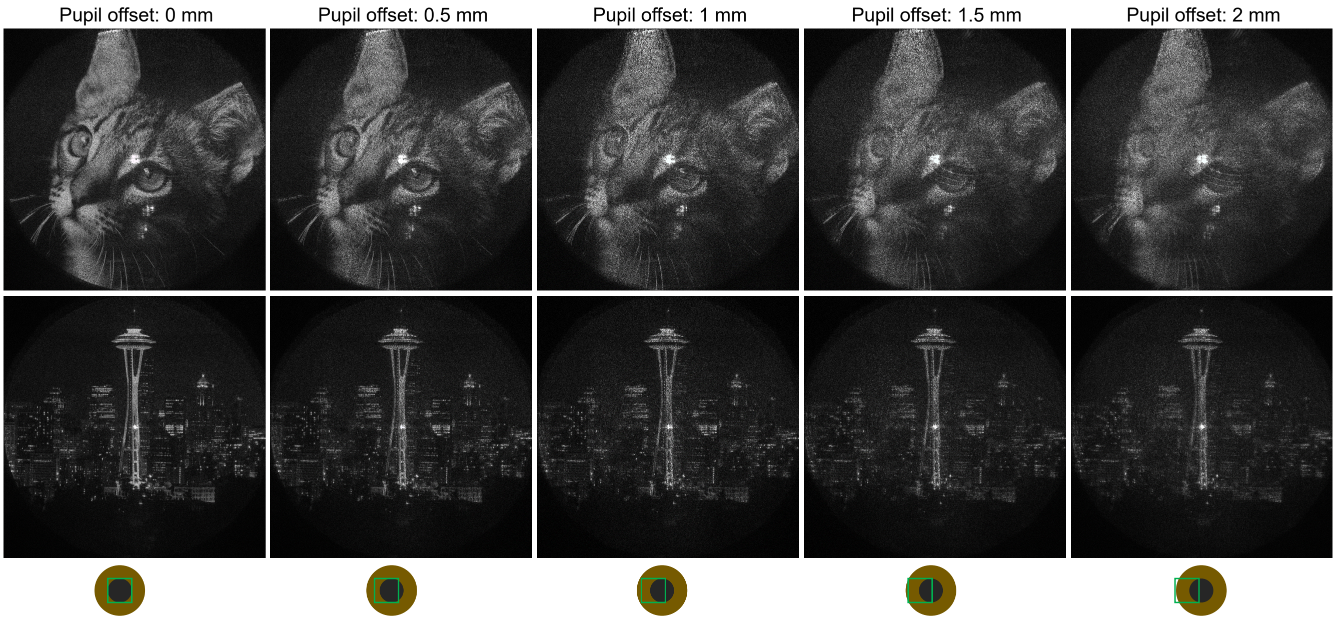

Supplementary Figure 14. Effect of pupil offset. Captured wavefront is cropped with different offset values and numerically propagated to simulate the image degradation with presence of eyetracking error. The optimized pupil size could be set larger than user's actual pupil size to provide a margin for eyetracking error. Cat image by Lali Masriera (CC BY 2.0).

Supplementary References

1. Levola, T. 7.1: Invited paper: Novel diffractive optical components for near to eye displays. In *SID Symposium Digest of Technical Papers*, vol. 37, 64–67 (Wiley Online Library, 2006).
2. Levola, T. Diffractive optics for virtual reality displays. *J. The Soc. for Inf. Disp. - J SOC INF DISP* **14**, 10.1889/1.2206112 (2006).
3. Kress, B. & Shin, M. Diffractive and holographic optics as optical combiners in head mounted displays. In *Proceedings of the 2013 ACM conference on Pervasive and ubiquitous computing adjunct publication*, 1479–1482 (2013).
4. Kress, B. C. & Cummings, W. J. 11-1: invited paper: towards the ultimate mixed reality experience: Hololens display architecture choices. In *SID symposium digest of technical papers*, vol. 48, 127–131 (Wiley Online Library, 2017).
5. Mukawa, H. *et al.* A full-color eyewear display using planar waveguides with reflection volume holograms. *J. society for information display* **17**, 185–193 (2009).
6. Han, J., Liu, J., Yao, X. & Wang, Y. Portable waveguide display system with a large field of view by integrating freeform elements and volume holograms. *Opt. express* **23**, 3534–3549 (2015).
7. Weng, Y. *et al.* Liquid-crystal-based polarization volume grating applied for full-color waveguide displays. *Opt. Lett.* **43**, 5773–5776 (2018).
8. Yoo, C., Bang, K., Chae, M. & Lee, B. Extended-viewing-angle waveguide near-eye display with a polarization-dependent steering combiner. *Opt. Lett.* **45**, 2870–2873 (2020).
9. Yoo, C. *et al.* Dual-focal waveguide see-through near-eye display with polarization-dependent lenses. *Opt. Lett.* **44**, 1920–1923 (2019).
10. Afra, T., Salehi, M. & Abiri, E. Design of two compact waveguide display systems utilizing metasurface gratings as couplers. *Appl. Opt.* **60**, 8756–8765 (2021).
11. Wyrowski, F. & Bryngdahl, O. Digital holography as part of diffractive optics. *Reports on Prog. Phys.* **54**, 1481 (1991).
12. Lee, S. *et al.* Viewing angle enhancement of an integral imaging display using bragg mismatched reconstruction of holographic optical elements. *Appl. Opt.* **55**, A95–A103 (2016).
13. Pytorch. https://pytorch.org/. Accessed on September 30, 2022.

14. Liebmann, M., Valverde, J. & Kerbstadt, F. Wavefront compensation for spatial light modulators based on Twyman-Green interferometry. In Lee, J.-H., Wang, Q.-H. & Yoon, T.-H. (eds.) *Advances in Display Technologies XI*, vol. 11708, 117080X, 10.1117/12.2583077. International Society for Optics and Photonics (SPIE, 2021).

15. Fan-Chiang, K.-H., Wu, S.-T. & Chen, S.-H. Fringing-field effects on high-resolution liquid crystal microdisplays. *J. Disp. Technol.* **1**, 304 (2005).

16. Piecewise affine transformation. https://scikit-image.org/docs/stable/auto_examples/transform/plot_piecewise_affine.html. Accessed on September 30, 2022.

17. Matsushima, K. & Shimobaba, T. Band-limited angular spectrum method for numerical simulation of free-space propagation in far and near fields. *Opt. Express* **17**, 19662–19673, 10.1364/OE.17.019662 (2009).

18. Matsushima, K., Schimmel, H. & Wyrowski, F. Fast calculation method for optical diffraction on tilted planes by use of the angular spectrum of plane waves. *J. Opt. Soc. Am. A* **20**, 1755–1762, 10.1364/JOSAA.20.001755 (2003).

19. Mantiuk, R., Kim, K. J., Rempel, A. G. & Heidrich, W. Hdr-vdp-2: A calibrated visual metric for visibility and quality predictions in all luminance conditions. *ACM Trans. Graph.* **30**, 10.1145/2010324.1964935 (2011).

20. Matsushima, K. *Introduction to Computer Holography: Creating Computer-Generated Holograms as the Ultimate 3D Image*. Series in Display Science and Technology (Springer International Publishing, 2020).

21. Li, X., Liu, J., Jia, J., Pan, Y. & Wang, Y. 3d dynamic holographic display by modulating complex amplitude experimentally. *Opt. Express* **21**, 20577–20587, 10.1364/OE.21.020577 (2013).

22. Bang, K., Jang, C. & Lee, B. Compact noise-filtering volume gratings for holographic displays. *Opt. Lett.* **44**, 2133–2136, 10.1364/OL.44.002133 (2019).

23. Kim, Y.-H. *et al.* Development of high-resolution active matrix spatial light modulator. *Opt. Eng.* **57**, 061606 (2018).

24. Hwang, C.-S. *et al.* 21-2: Invited paper: 1μm pixel pitch spatial light modulator panel for digital holography. In *SID Symposium Digest of Technical Papers*, vol. 51, 297–300 (Wiley Online Library, 2020).

25. Xiong, Y. *et al.* Coherent backlight system for flat-panel holographic 3d display. *Opt. Commun.* **296**, 41–46 (2013).

Acknowledgements

In Supplementary Fig. 4, the bicycle image by Fiore Power (CC BY 2.0). In Supplementary Fig. 8, the cat image by Lali Masriera (CC BY 2.0), the Seattle skyline image by fiction-parade (CC BY-SA 2.0). In Supplementary Fig. 11 - 13, the bus image (second row) by Farhad Sadykov (CC BY 2.0), the house image (5th row) by Patrick Feller (CC BY 2.0), the Tudor house image (9th row) by Nikk (CC BY 2.0). The research is supported by Meta.
