# Supplementary material for: Waveguide holography for 3D augmented reality glasses
Source: Nat Commun. 2024 Jan 2;15:66. doi: 10.1038/s41467-023-44032-1 (PMC10762208; doi:10.1038/s41467-023-44032-1)
Supplement: Supplementary file 3 — Description of Additional Supplementary Information [file 41467_2023_44032_MOESM3_ESM.pdf]

Description of Additional Supplementary Files:

Supplementary Movie 1: Continuous focus change of full 3D result. Robot images are rendered by Tech Art team in Meta.
